# Current concepts in hand infections

**DOI:** 10.1186/1753-6561-9-S3-A102

**Published:** 2015-05-19

**Authors:** Tolga Turker

**Affiliations:** 1 Department of Surgery, University of Arizona, Tucson, Arizona, 85721, USA

## 

Hand infections are common occurrences, usually resulting from an injury, that when left untreated can quickly lead to tissue destruction and loss of function or permanent disability. Infections may be categorized anatomically: superficial, involving the tendon and tendon sheath, involving joint or bone, or affecting the deep spaces of the hand. Infections may be caused by different microorganisms, and, increasingly, by community-acquired methicillin-resistant *Staphylococcus aureus* (MRSA). Currently MRSA accounts for as much as 65% of *Staphylococcus aureus* isolates, complicating the course of medical treatment. Brown and Young state that major metropolitan hospitals should expect 25-50 admissions annually for serious hand infection. Hand infections primarily occur after delayed treatment after minor trauma. Laceration, unknown causation, thorn, human bite (e.g., often “fight-bite” injuries), IV injection injury, dog bite, insect bite, blunt trauma, cat bite, snake bite, and pressure injections. The palm or dorsum of the hand, the middle finger and the index finger are most frequent locations of infection. It is reported that the most common hand infections are cellulitis and paronychia/eponychia (70%) and more severe infections such as septic arthritis and osteomyelitis occur less often (3%).

A wide variety of approaches to antibiotic treatment have been used and evolved over the years as antibiotic resistance and occurrence of community-acquired antibiotic-resistant infections have increased. Even though the choice of antibiotics is evolving from those used in the past due to changes in microorganisms and developing antibiotic resistance, the treatment principles set forth by Brown and Young of 3-5 days of IV antibiotic treatment followed by 7-10 days of oral antibiotic treatment remains a valid treatment plan for hand infection patients.

Another important issue in hand infections is delay in seeking treatment and/or delayed surgical drainage. Glass reported that delay in treatment causes slower resolution; for example, if the delay is more than 2.5 days, about 70% of those patients showed delayed recovery.

Surgical treatment is an important component of clinical management. A detailed, comprehensive initial surgical approach may minimize the need for multiple surgeries. Intraoperative care should be comprehensive, using copious Dakin’s solution, hydrogen peroxide, sterile water, and bacitracin in normal saline to irrigate the infected area. Postoperatively, usage of Dakin’s solution for immediate soaking of the open wound 1-2 times per day for 10 minutes for 3 days may help to decrease the load of bacteria. Although traditionally, post-operative splinting may be suggested for hand infection treatment, we recommend application of a soft dressing and starting early range of motion exercises in order to decrease stiffness.

In conclusion, with prompt and appropriate care, most soft tissue hand infection patients can achieve full resolution of their infections. Treating infections promptly with surgical incision and drainage using a large incision with copious irrigation and then using a regimen of soaking and use of soft dressing with early range of motion exercises are key to achieving good outcomes and avoiding multiple surgical procedures. In conjunction with surgical treatment, judicious antibiotic treatment is also important. After cultures are obtained, antibiotic treatment should be tailored more specifically to the organisms. Consultation with an infectious diseases specialist can sometimes be helpful in choosing the most appropriate regimen.
